# Hapten Synthesis and the Development of an Ultrasensitive Indirect Competitive ELISA for the Determination of Diethylstilbestrol in Food Samples

**DOI:** 10.1038/s41598-020-59112-1

**Published:** 2020-02-24

**Authors:** Xingdong Yang, Yinbiao Wang, Chunmei Song, Xiaofei Hu, Fangyu Wang, Xianyin Zeng

**Affiliations:** 1 Institute of Food and Drug Inspection, Zhoukou Normal University, Zhoukou, 466001 P.R. China; 2 Key Laboratory of Animal Immunology of the Ministry of Agriculture, Henan Provincial Key Laboratory of Animal Immunology, Henan Academy of Agricultural Sciences, Zhengzhou, 450002 P.R. China; 3 Department of Veterinary science, college of Life science, Sichuan Agricultural University, Ya’an, 625014 P.R. China; 4 School of Public Health, Xinxiang Medical University, Xinxiang, 453003 P.R. China; 5 Food and Bioengineering College, Xuchang University, Xuchang, 461000 P.R. China

**Keywords:** Assay systems, Applied immunology

## Abstract

An ultrasensitive indirect competitive enzyme-linked immunosorbent assay (*ic* ELISA) using monoclonal antibodies (mAbs) was developed for the specific detection of diethylstilbestrol (DES) residues. To establish an ELISA based on mAbs, hapten diethylstilbestrol mono-carboxypropyl-ether (DES-MCPE) was chemically synthetized and then conjugated to bovine serum albumin (BSA) for immunization in mice. This *ic* ELISA was further optimized for DES determination. The sensitivity of the *ic* ELISA was found to be 0.49 *μ*g/kg and the limit of detection was 0.075 *μ*g/kg. DES residues in salmon meat and pork were tested with the recovery range from 74.0 to 85.2% and the coefficient of variation (CV) was less than 10%. Parallel analysis of DES samples from salmon meat showed comparable results from the *ic* ELISA with high-performance liquid chromatography. The *ic* ELISA provides a useful screening method for the quantitative detection of DES residues in animal-derived food.

## Introduction

Diethylstilbestrol (DES; 4,4’-(3E)-hex-3-ene-3,4-diyldiphenol), also known as Stilbestrol, is a synthetic oestrogen and can have the same pharmacological and therapeutic effects as natural oestradiol. DES has been mainly used in the clinical treatment of oestrogen-deficiency disorders, functional bleeding and amenorrhea and can also be used to improve the sensitivity of myometrium to oxytocin in stillbirth odynopoeia^1,2^. However, DES was widely used in animal production as a growth promoting additives for increasing animal lean meat percentage and feed efficiency^3^. Currently, in order to maximize benefits, DES is still illegally used by some farmers from time to time in China. Due to the accumulation and long half-life of DES residues in the human body via the food chain, DES can cause abnormalities in the human reproductive system, infertility, pregnancy complications, increased incidence of breast cancer and vaginal clear-cell carcinoma in women^4–9^. Consequently, the use of DES in animals has been banned in many countries.

Specific and sensitive methods for detecting DES have been developed. Classical methods for the determination of DES include high-performance liquid chromatography (HPLC)^10^, ultra-HPLC tandem mass spectrometry (UHPLC- MS/MS)^11,12^, and gas chromatography tandem mass spectrometry (GC-MS/MS)^13,14^. Although these methods are highly accurate, they do require extensive sample preparation, expensive instruments, and professionals to operate. Recently, an enzyme-linked immunosorbent assay (ELISA) was shown to meet the needs of analysis for DES with easy operation, cost effectiveness and less required time; ELISAs have been increasingly used for analysing haptens such as drugs^15,16^, pesticides^17,18^, toxins^19^, and allergen^20^ residues.

However, because of the lack of groups for direct coupling to carrier proteins, there have been few reports on the establishment of an antibody-based ELISA against DES. Xu *et al*. synthesized an antigen of DES-MCPE-BSA and developed a competitive ELISA based upon monoclonal antibodies (mAbs) for determining DES in chicken and livers tissues samples^21^. Zhang *et al*. developed an indirect competitive ELISA to quantify DES in human urine^22^. The 50% inhibition of antibody binding (IC_50_) of these two methods were 2.4 and 3.33 *μ*g/kg, respectively, and meet the maximum residue limits (MRLs) (<1.5 *μ*g/kg) in animal food of the European Union, United States, and China, etc. In this paper, we described the development of an *ic* ELISA for the rapid detection of DES residues in animal muscle tissues using a mAb 4C7, specific for DES. The IC_50_ of the *ic* ELISA was 0.49 *μ*g/kg. The immunoassay is suitable for the screening of large numbers of animal-derived food samples.

## Materials and Methods

### Reagents and materials

1-(3-(dimethylamino) propyl)-3-ethylcarbodiimide hydrochloride (EDC), N-hydroxy succinimide (NHS), DES, freund’s adjuvants and the mouse mAbs isotyping kit were bought from Sigma (St. Louis, MO, USA). Bovine serum albumin (BSA) and ovalbumin (OVA) were purchased from Yuanye Biotechnology Co., Ltd. (Shanghai, China). HRP conjugated goat-anti-mouse IgG antibody was obtained from Sino-American Biotechnology Co., Ltd (Luoyang, China).

### Hapten synthesis

A derivative of DES, called DES-MCPE, was chemically synthetized using ethyl 4-bromobutyrate as follows. Fifty-four milligrams of DES was dissolved in 2 mL of anhydrous dimethyl sulfoxide (DMSO), then 27.5 mg of potassium carbonate was added, and the mixture was stirred at room temperature for 1.5 h in the dark. Seventeen milligrams of ethyl 4-bromobutyrate was added to the mixture and stirred at room temperature for 8 h in the dark. The reaction product was transferred to 10 mL of pre-cooled dilute hydrochloric acid solution (pH 2), and 5 mL of ethyl acetate was added with double distilled water for extraction. The extracts were rinsed with double distilled water and placed into a vacuum concentrator (jouan-rc1010z, France) to extract the organic solvent. The extracted product was dissolved in 2 mL of methanol solution, and then 2 mol/L sodium hydroxide solution was added. Once the product was fully dissolved, an appropriate amount of hydrochloric acid solution was added dropwise to maintain the pH value of the solution between 2 to 4. The above steps were repeated, and the product was extracted by ethyl acetate, rinsed with double distilled water, and vacuum dried to obtain the DES-MCPE.

### Hapten-carrier protein conjugation

The hapten of DES-MCPE was conjugated to BSA or OVA by the EDC/NHS method^23^. Forty-two milligrams of DES-MCPE was dissolved in 2 mL of dimethyl sulfoxide, and 25 mg of EDC, 0.6 mL of anhydrous dimethylformamide, and 15 mg of NHS were added to the mixture. The mixture was stirred at room temperature for 4 h in the dark and centrifuged at 2900 g for 5 min to collect the supernatant. Sixty milligrams of BSA was fully dissolved in a mixture of 1 mL of anhydrous dimethylformamide and 2 mL phosphate buffer saline (PBS) (PH 8). In ice bath conditions, the supernatant was added dropwise to the BSA solution and stirred at 4 °C for 8 h. The reaction product was dialysed against PBS (PH 7.2) for 8 h.

### Preparation of monoclonal antibodies

For the DES immunogen, subcutaneous injections were given to three female BALB/c mice (at the animal experimental centre of Zhengzhou University, Zhengzhou, China). For the first immunization, 0.2 mL of Freund’s complete adjuvant was emulsified with the conjugate (60 *μ*g of immunogen in 0.2 mL of PBS) (1:1, v/v) for injection into a mouse weighing 15~22 g. At intervals of 21 days, Freund’s incomplete adjuvant was used for fortified subcutaneous immunization. Ten days after the fourth immunization, 10 *μ*L of blood sample from each mouse was obtained, and the collected antiserum was evaluated by indirect competition ELISA (*ic* ELISA). Sp2/0 myeloma cells were fused with the splenocytes of the selected mouse using PEG-1500 72 h after intraperitoneal injection of 100 *μ*g of immunogen in 150 *μ*L of PBS buffer. Ten days later, hybridomas were selected by *ic* ELISA, and lowest value of IC_50_ were considered positive, subcloning was performed by the limited dilution method. The ascitic fluids of positive hybridomas were produced in mice with liquid paraffin in the peritoneal cavity.

### Development of the ic ELISA

An *ic* ELISA was developed according to the description of Sun *et al*.^24^. Serum samples were screened for polyclonal or monoclonal antibodies specific to DES using an *ic* ELISA. Antigen for detection (2 *μ*g/mL OVA conjugate in carbonate buffer, 50 *μ*L well^−1^) was coated in 96-well ELISA plates at 37 °C for 2 h. Then, each well was washed four times with phosphate buffered solution [PBST, PBS + 0.05% Tween-20 (V:V)] and blocked with 220 *μ*L/well of 5% pig serum in PBST at 37 °C for 60 min, and the plates were washed four times with PBST and dried at room temperature. Fifty microlitres of diluted DES in PBS was added to each well, and 50 μL of diluted antibody solution was added. After 20 min in a constant temperature incubator at 37 °C, the plate was washed four times with PBST, and goat anti-mouse IgG labelled horseradish peroxidase (GAMIgG-HRP) was diluted 1:1000 and added at 50 *μ*L/well. 3, 3’, 5, 5’-Tetramethylbenzidine (TMB) colour liquid was added to each well and incubated until the colour development of each well was ideal. Finally, 50 *μ*L of 2 mol/L sulfuric acid was added to terminate the reactive liquid and the absorbance values of the optical density at a wavelength of 450 nm (OD_450nm_) were mensurated using a microplate reader 550 (Bio-Rad, Richmond, CA, USA).

### Optimization of the ic ELISA

To improve the sensitivity of the *ic* ELISA, we optimized different parameters, including the antibody-working concentration, coating concentration, coating time, blocking solution, diluted concentration of GAMIgG-HRP, and time of TMB colorization. Concentrations of the working antibody and coating antigen were optimized by checkerboard titration using DES-MCPE-OVA as the coating antigen^25^. Briefly, the DES-MCPE-OVA was coated on the ELISA plate at concentrations of 0.1, 0.2, 0.5, 1.0, 2.0, and 4.0 *μ*g/mL and the mAbs towards DES were diluted with PBS at dilutions of 1:4.0 × 10^3^, 1:8.0 × 10^3^, 1:1.6 × 10^4^, 1:3.2 × 10^4^, 1:6.4 × 10^4^, 1:1.28 × 10^5^, 1:1.28 × 10^5^, and 1:5.12 × 10^5^. The OD_450nm_ value was obtained by microplate reader. A well was selected when its OD _450 nm_ value was approximately 1.0, and the difference of its OD_450nm_ value was significant relative to its adjacent wells. The concentrations of DES-MCPE-OVA and anti-DES mAbs corresponding to this well were considered optimal. For other parameters, the immunoassay performance was evaluated using the Amax (maximal absorbance)/IC_50_ ratio. ELISA sensitivity is positively correlated with the Amax/IC_50_ ratio^26^.

### Sample preparation

DES-negative salmon meat and pork were purchased from a local supermarket, and both were analysed by LC-MS. Eight-grams of salmon meat was cut into small pieces, homogenized using a high-speed homogenizer, and 16 mL of methanol was added. The mixture was shaken on a vortex oscillator for 25 min and centrifuged for 9 min at 3300 g. The supernatant was filtered and concentrated to 8 ml. The negative pork was also processed using the same operation.

### Characterization of the ic ELISA

Cross reactivity (CR) was used to identify the specificity of the *ic* ELISA. Sample of DES and structural analogues (Hexestrol, Diethylstilbestrol, Progesterone, Oestradiol, Bisphenol A and Estriol) were added to salmon meat samples at a concentration of 1000 *μ*g/kg. The calculation formula for CR: CR (%) = (IC_50_ of DES/IC_50_ of the structural analogues) × 100%^27^.

To estimate the accuracy of the *ic* ELISA, salmon meat and pork samples including the DES standard (2.0, 10.0, and 50.0 *μ*g/kg) were detected via *ic* ELISA in sextuplicate. Recovery rate and coefficient of variation (CV) were used to illuminate accuracy.

### HPLC Confirmation

Samples of salmon meat including three different levels of DES (8.0, 16.0, 32.0 *μ*g/kg), were tested in sextuplicate by *ic* ELISA and HPLC. One sample *t* test was used to analyse the measured data for the difference between *ic* ELISA and HPLC methods.

## Results and Discussion

### Design of haptens and characterization of DES conjugates

**Figure 1:**
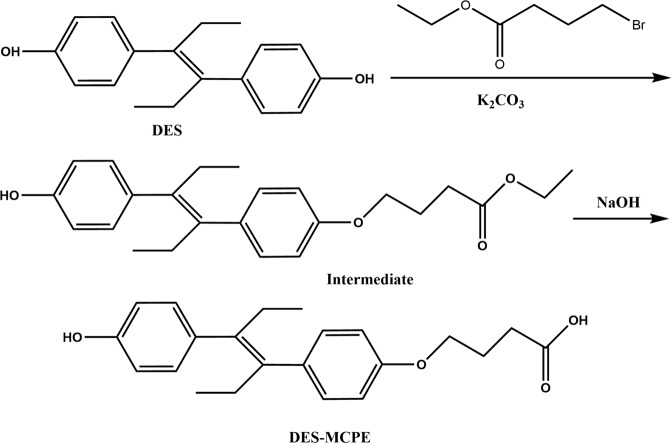
The synthetic routes of DES-MCPE.

**Figure 2:**
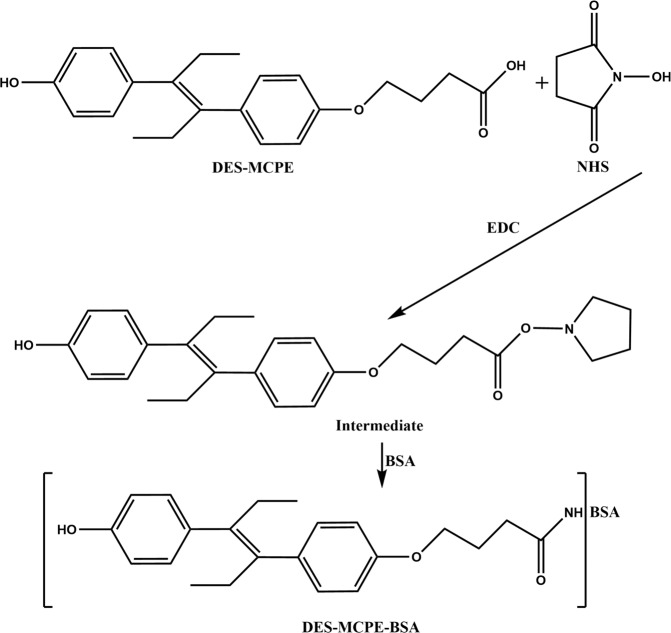
The synthetic routes of DES-MCPE-BSA.

For developing an immunoassay to detect DES, it is very important to synthesize effective haptens to produce an anti-DES antibody. A derivative of DES, called DES-MCPE (Fig. 1), was chemically synthesized using ethyl 4-bromobutyrate, which can be conjugated to BSA using the EDC/NHS method (Fig. 2). Coupling ratios of DES-MCPE to BSA and OVA were 12.8:1 and 11.5:1, respectively.

In this work, the antigen was synthesized 6 times to be successful, and the antiserum of mice was not sensitive against DES when the hapten had not been purified, so synthesizing the antigen is meticulous work. To allow efficient conjugation, a spacer arm including 4–6 carbons has been reported to be optimal^28^. A spacer group containing 4 carbons was conjugated with DES for complete antigen preparation. The reactivities of polyclonal antiserum are not ideal when testing after the fourth immunization which might be caused by low conjugation rate or loss of antigen emulsification. The mice were immunized another time with DES-MCPE-BSA to obtain sensitive, specific antibodies for developing an immunoassay based on DES-MCPE-BSA to detect DES. We also tried to synthesize another antigen, DES-hemisuccinate (DES-HS), which was chemically modified via the succinic anhydride method of Hongsibsong *et al*.^29^ and then conjugated to BSA by the mixed anhydride method of Wainer *et al*.;^30^ the coupling ratios of DES-HS to BSA and OVA were 9.4:1 and 7.6:1, respectively. However, DES-HS-BSA elicited antibodies which showed higher potency in indirect ELISA (>1:128000), but poor reactivity with DES in *ic* ELISA.

### Production and characteristic of mAbs

After cell fusion, the supernatant of each hybridomas was tested using indirect ELISA and *ic* ELISA. Four hybridomas 1B8, 2C4, 4A1, and 4C7, gave the best results in both ELISAs and were used to prepare ascites in mice. Anti-DES titres of the IgGs extracted from the ascites of 1B8, 2C4, 4A1, and 4C7 were 1:2.0 × 10^5^, 1:2.56 × 10^5^, 1:1.28 × 10^5^, and 1:5.12 × 10^5^ respectively. The affinity constant (Ka) for each mAb reached 3.38 × 10^9^, 9.27 × 10^9^, 2.30 × 10^9^, and 1.87 × 10^10^ L/mol, respectively. mAb 4C7 gave the best IC_50_ value of 0.49 μg/kg and was used in the *ic* ELISA.

### Development and optimization of ic ELISA

**Figure 3:**
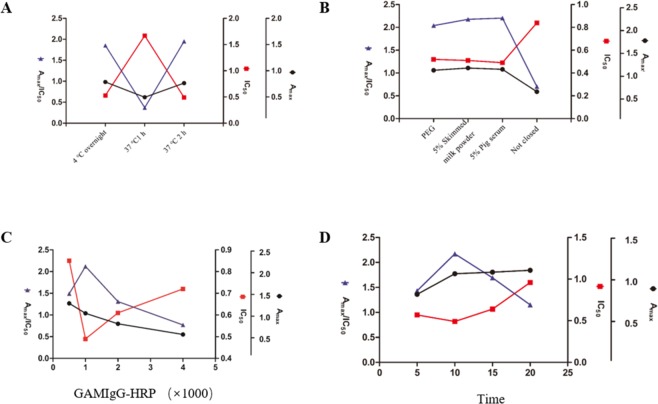
Optimization of experimental conditions on the *ic* ELISA performances.

An *ic* ELISA was established using the mAbs 4C7, based on the competitive binding of free DES in the sample and coated DES-MCPE-OVA. The optimal conditions for the *ic* ELISA were determined by chessboard ELISA as follows: the diluted concentration of the mAb was 1:6.4 × 10^4^, and the coating concentration of DES-MCPE-OVA was 0.5 *µ*g/mL. For other parameters, the highest Amax /IC_50_ ratio was selected; for example, the ELISA plate was coated with DES-MCPE-OVA at 37 °C for 120 min (Fig. 3A), blocked for 60 min with 5% porcine serum at room temperature (Fig. 3B), the diluted concentration of GAMIgG- HRP was 1:1 × 10^3^ (Fig. 3C), and TMB was used for colour development at room temperature for 10 min (Fig. 3B).

### Sensitivity, specificity and reproducibility of the ic ELISA

**Figure 4:**
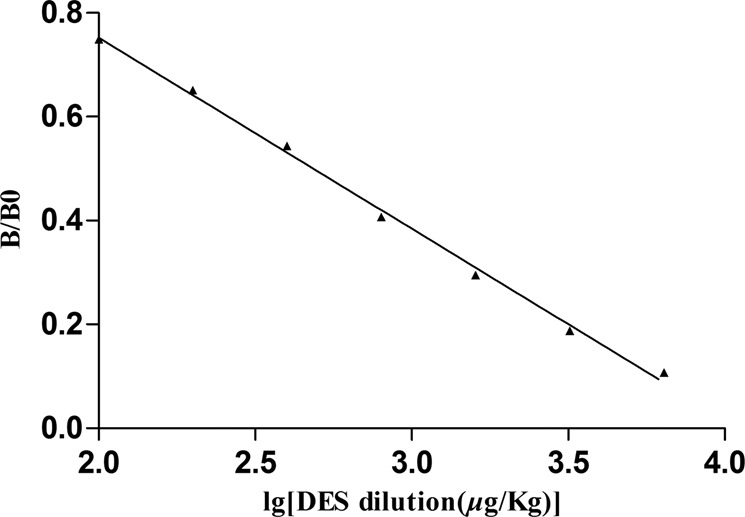
Standard curve plotted from the values of B/B_0_ against the logarithmic concentrations of DES.

According to the optimized reaction conditions, reference solutions of DES at concentrations of 0, 0.1, 0.2, 0.4, 0.8, 1.6, 3.2, and 6.4 μg/kg were analysed using the *ic* ELISA. Assays were performed in triplicate, and typical results are shown in Fig. 4. The immunoassay displayed an IC_50_ of 0.49 *μ*g/kg, and the limit of detection was 0.075 *μ*g/kg for DES determination.

DES and structural analogues, including hexestrol, dienestrol, progesterone, oestradiol, bisphenol A and estriol were added to salmon meat samples and tested. The *ic* ELISA gave 100%, 7.66% and 3.83% CR for DES, hexestrol and dienestrol, respectively, and produced no CR with other compounds (<0.01%) including progesterone, oestradiol, bisphenol A and estriol (Table 1).

**Table 1:** CR of the ic ELISA for detecting DES and its analogues.

Compounds	structures	IC_50_ (*μ*g/kg)	CR (%)
Diethylstilbestrol		0.49	100
Hexestrol		6.4	7.66
Dienestrol		12.8	3.83
Progesterone		>1.0 × 10^3^	< 0.01
Oestradiol		>1.0 × 10^3^	< 0.01
Bisphenol A		>1.0 × 10^3^	< 0.01
Estriol		>1.0 × 10^3^	< 0.01

To determine the reproducibility of the *ic* ELISA, salmon meat and pork samples containing 2.0, 10.0, and 50.0 *μ*g/kg DES were tested using *ic* ELISA. The recoveries ranged from 74.0 to 89.6% for reproducibility. The CVs of the *ic* ELISA were all less than 10.0% (Table 2).

**Table 2:** Accuracy of the ic ELISA for detecting DES in salmon meat and pork samples.

Food samples	Spiked DES (*μ*g/kg)	Mean±SD (*μ*g/kg)	Recovery (%)	CV (%)
salmon meat	2.0	1.48 ± 0.12	74.0 ± 6.0	8.1
	10.0	7.46 ± 0.74	74.6 ± 7.40	9.9
	50.0	41.49 ± 4.01	82.7 ± 8.06	9.7
pork	2.0	1.49 ± 0.11	74.5 ± 5.50	7.3
	10.0	7.72 ± 0.71	77.2 ± 7.10	9.2
	50.0	42.6 ± 3.65	85.2 ± 7.30	8.6

### Comparison between ic ELISA and HPLC

The performance of the *ic* ELISA was validated by HPLC at three levels with authentic samples. Concentrations of DES in the salmon meat at 8.0, 16.0, and 32.0 *μ*g/kg were determined using an *ic* ELISA and HPLC. Statistical analysis using one sample t-test did not show a significant difference between *ic* ELISA and HPLC (Table 3).

**Table 3:** Comparison of the *ic* ELISA test with HPLC using three levels of DES residues in salmon meat.

DES concentration (*μ*g/kg)	*ic* ELISA (*μ*g/kg)	HPLC (*μ*g/kg)
8.0	6.07 ± 0.55	6.69 ± 0.31
16.0	12.52 ± 1.08	13.60 ± 0.73
32.0	25.64 ± 2.37	27.65 ± 1.91

## Conclusion

An *ic* ELISA was established using a high-affinity, specific mAb, 4C7, against DES for the detection of DES residues. Upon optimization, the IC_50_ was found to be 0.49 *μ*g/kg, and the limit of detection was 0.075 *μ*g/kg in the reference solutions. Recovery from salmon meat and pork samples was tested and found to range from 74.0 to 85.2%. The major advantages of the *ic* ELISA are that it is relatively more cost-effective and requires a shorter time than chromatographic instrument analysis, and it is easy to operate by professionals. Therefore, the *ic* ELISA has the potential to be used as a rapid screening tool for detecting DES residues in animal-derived food samples.

### Ethical statement

All BALB/c mice in this experiment were approved by the animal ethics committee of Zhoukou Normal University (approval No. ZKNU-1-2018022701-1002) and were used in accordance with all applicable institutional and governmental regulations concerning the ethical use of animals. This article does not contain any studies with human participants performed by any of the authors.
